# Biomarker-driven trial in metastatic pancreas cancer: feasibility in a multicenter study of saracatinib, an oral Src inhibitor, in previously treated pancreatic cancer

**DOI:** 10.1002/cam4.27

**Published:** 2012-08-16

**Authors:** John Arcaroli, Kevin Quackenbush, Arvind Dasari, Rebecca Powell, Martine McManus, Aik-Choon Tan, Nathan R Foster, Joel Picus, John Wright, Sujatha Nallapareddy, Charles Erlichman, Manuel Hidalgo, Wells A Messersmith

**Affiliations:** 1 University of Colorado Cancer CenterDenver, Colorado; 2 University of Texas MD Anderson Cancer CenterHouston, Texas; 3 Mayo Clinic Cancer CenterRochester, Minnesota; 4 Washington University School of MedicineSt. Louis, Missouri; 5 Cancer Therapy Evaluation ProgramBethesda, Maryland; 6 Centro Nacional de Investigaciones OncológicasMadrid, Spain

**Keywords:** Biomarker, clinical trial, pancreas cancer, Src

## Abstract

Src tyrosine kinases are overexpressed in pancreatic cancers, and the oral Src inhibitor saracatinib has shown antitumor activity in preclinical models of pancreas cancer. We performed a CTEP-sponsored Phase II clinical trial of saracatinib in previously treated pancreas cancer patients, with a primary endpoint of 6-month survival. A Simon MinMax two-stage phase II design was used. Saracatinib (175 mg/day) was administered orally continuously in 28-day cycles. In the unselected portion of the study, 18 patients were evaluable. Only two (11%) patients survived for at least 6 months, and three 6-month survivors were required to move to second stage of study as originally designed. The study was amended as a biomarker-driven trial (leucine rich repeat containing protein 19 [LRRC19] > insulin-like growth factor-binding protein 2 [IGFBP2] “top scoring pairs” polymerase chain reaction [PCR] assay, and PIK3CA mutant) based on preclinical data in a human pancreas tumor explant model. In the biomarker study, archival tumor tissue or fresh tumor biopsies were tested. Biomarker-positive patients were eligible for the study. Only one patient was PIK3CA mutant in a 3′ untranslated region (UTR) portion of the gene. This patient was enrolled in the study and failed to meet the 6-month survival endpoint. As the frequency of biomarker-positive patients was very low (<3%), the study was closed. Although we were unable to conclude whether enriching for a subset of second/third line pancreatic cancer patients treated with a Src inhibitor based on a biomarker would improve 6-month survival, we demonstrate that testing pancreatic tumor samples for a biomarker-driven, multicenter study in metastatic pancreas cancer is feasible.

## Introduction

Pancreatic adenocarcinoma is the fourth leading cause of cancer deaths annually. In 2011, the estimated incidence was 44,030 with 37,660 deaths [1]. Although there is a better understanding of molecular events and newer therapies for pancreatic cancer, the 5-year survival rate still remains extremely poor at only 4% [2]. Gemcitabine is the standard therapy in the first-line setting, but only improves the median survival to 5.65 months when compared with 5-fluorouracil [3]. Recently, a randomized phase III trial of FOLFIRINOX (F: 5FU/leucovorin [LV], irinotecan [I], and oxaliplatin [O]) as first-line treatment for metastatic pancreatic adenocarcinoma showed a 4.3-month improvement in overall survival when compared with gemcitabine, but at a price of higher toxicity [4]. Most targeted agents have failed to provide any meaningful improvement in outcome. The continued poor survival despite the new understanding of pancreatic cancer biology and the incorporation of novel therapies demonstrates an acute need for improvement in therapy. In addition, given the heterogeneity of the genetic background of pancreas cancer, biomarker-driven strategies in patient subsets may be needed to advance the field, but the quantity and quality of diagnostic tissue is often low.

One target is the nonreceptor kinase c-Src [5]. This protein has been shown to regulate many cellular events that modulate cellular proliferation, adhesion, migration, and invasion by activating downstream targets such as focal adhesion kinase (FAK), paxillin (PAX), and signal transducer and activator of transcription 3 (STAT-3) [6–10]. The dysregulation of Src has been implicated in the development and progression of many human malignancies [11–15]. In pancreatic cancer, inhibition of Src activity has been shown to have antitumor and antimetastatic activity. In a pancreas orthotopic model, Src inhibition alone and in combination with gemcitabine reduced tumor burden and the number of lymph node and liver lesions [16]. We demonstrated in a patient-derived pancreatic adenocarcinoma xenograft model that treatment with saracatinib (a Src inhibitor) decreased tumor growth in a subset of tumors [17]. By comparing sensitive and resistant tumors, a gene “top scoring” pair leucine rich repeat containing protein 19 (LRRC19) gene expression > insulin-like growth factor-binding protein 2 (IGFBP2) gene expression achieved high accuracy in predicting sensitivity preclinically [17]. Upregulation of LRRC19 has been shown to occur in hypoxic conditions [18], whereas IGFBP2 has been demonstrated to play a role in the development and progression of gliomas [19].

The PI3K/Akt signaling pathway plays an integral role at enhancing cellular survival and proliferation [20–22]. Mutations in the PIK3CA gene exon 9 (helical) and 20 (kinase) have been identified in many different cancers [23] and appear to be important in tumorigenesis. In pancreas cancer, the frequency is low with approximately 3% of patients harboring a common PIK3CA mutation (542, 545, or 1047) [24]. Interestingly, recent work has shown that Src interacts with the PI3K pathway. In particular, enhanced Src activity diminishes PTEN stability and therefore facilities an increase in AKT activation [25]. We have found that Src interacts with the PI3K regulatory subunit p85 to yield an increase in Akt activation (manuscript submitted). Therefore, Src inhibition may be beneficial in tumors with activating mutations in PIK3CA or in tumors dependent on the PI3K pathway.

Clinical trials involving Src inhibitors as monotherapy have been investigated in many different tumor types [26]. Although these trials have failed to find benefit in an unselected population, biomarkers of sensitivity or resistance may enrich for those patients that likely would derive benefit from these compounds. As preclinical studies indicate a role of Src in pancreatic cancer and that a biomarker may predict response to the Src inhibitor saracatinib, the Phase II Consortium (P2C) conducted a phase II clinical and biological study of saracatinib, an oral Src inhibitor, in gemcitabine-resistant metastatic pancreas cancer patients. The primary objective of the study was to determine the 6-month survival in patients treated with saracatinib in an unselected population. When a minimum number of patients achieving 6-month overall survival was not reached, the study was amended to determine if survival would be improved in biomarker-selected patients based on preclinical experiments with human tumor explants.

## Patients and Methods

### Eligibility criteria

Patients 18 years or older were eligible if they had histologically or cytologically confirmed metastatic pancreatic adenocarcinoma and had at least one prior regimen of chemotherapy, preferably gemcitabine based, as treatment for metastatic disease. Measurable disease was defined per RECIST version 1.0 [27]. Patients also had to have an ECOG performance status (PS) 0, 1, or 2 and adequate organ and marrow function. Exclusion criteria included women pregnant or nursing; patients who had not recovered from adverse events (excluding alopecia) due to agents (chemotherapy or radiotherapy) administered more than 4 weeks earlier; ongoing clinical requirement for administration of a strong inhibitor/inducer of CYP3A4; treatment with other investigational compounds; cardiac dysfunction including, but not limited to, symptomatic congestive heart failure, unstable angina pectoris, or cardiac arrhythmia; and HIV-positive patients on combination antiretroviral therapy.

The protocol was approved by NCI/CTEP as well as the institutional review boards of participating institutions, and written informed consent was obtained for all patients prior to performing study-related procedures in accordance with federal and institutional guidelines (http://clinicaltrials.gov).

### Treatment schedule

Treatment was administered on an outpatient basis. Patients took orally 175 mg daily (1 × 125-mg tablet plus 1 × 50-mg tablet). Treatment continued every day until disease progression, up to a maximum of 2 years from study entry. The cycle length was 4 weeks. Patients were provided with a Medication Diary, instructed in its use, and asked to bring it with them to each appointment. Dose modifications to 125 mg (dose level 1) or 100 mg (dose level 2) daily were allowed for grade >3/4 toxicities or investigator discretion.

### Clinical evaluation and safety assessment

Patients underwent history and physical examination, PS assessment and vital signs, complete blood count (CBC), chemistries, urinalysis, and tumor measurements within 14 days prior to the start of therapy and generally every cycle postbaseline. Chemistries were obtained at day 15 of cycle 1 as well. Serum pregnancy test (if applicable) and baseline tumor measurements (within 4 weeks of initiation of therapy) were also obtained. Adverse events were classified/graded weekly according to the Common Terminology Criteria of Adverse Events, version 3.0. Response was assessed every two cycles postbaseline per RECIST version 1.0.

### Biomarker: LRRC19 and IGFBP2

Biomarker studies were performed in Clinical Laboratory Improvement Amendments (CLIA)-approved laboratory space. Archival specimens were submitted for most subjects, but for patients without archival tissue, optional fresh biopsies (paid by the study) were done. Premade kits with reagents and labeled tubes were sent to participating institutions. Total RNA was extracted from archival tumor blocks or slides using the RNeasy FFPE kit (Catalog #73504 – Qiagen, Valencia, California) and fresh liver biopsies preserved in RNA*later* using the RNeasy mini kit (Catalog #74106 Qiagen) according to the manufacturer's instructions. Complementary DNA (cDNA) was synthesized using the Applied Biosystems (Foster City, California) high capacity cDNA reverse transcription kit, following the manufacturer's instructions. Validated and predesigned primer/probes for LRRC19 and IGFBP2 and the housekeeping gene UBC were purchased from Applied Biosystems. Samples were amplified using the LightCycler® 480 Real-Time PCR System in a CLIA-approved laboratory. The sensitive tumor panc 410 (LRRC19 > IGFBP2) and resistant tumor panc 198 (LRRC19 < IGFBP2) were used as assay controls on each plate. Relative expression of the mRNA analyzed was estimated using the formula: 2^−ΔCT^, where #*C*_T_ = *C*_T_ (mRNA) − *C*_T_ (Housekeeper). Any patient sample with the gene expression of LRRC19 greater than the gene expression of IGFBP2 was considered biomarker positive and eligible for clinical screening.

### Biomarker: PIK3CA

Total DNA was extracted from archival tumor or formalin-fixed fresh liver biopsies blocks or slides using the QIAamp DNA FFPE Tissue Kit (Catalog #56404 – Qiagen) according to the manufacturer's instructions. The DxS PIK3CA kit (Catalog #PK-02 – Qiagen) was used to determine the common mutations in the PIK3CA exon 9 (E542K, E545D, and E545K) and exon 20 (H1047R) according to the manufacturer's instructions. It is possible to detect approximately 1% mutant in the presence of wild-type background using this kit. Samples were amplified using the LightCycler® 480 Real-Time PCR System in a CLIA-approved laboratory. In addition to the PIK3CA DxS kit, PIK3CA exon 9 or 20 was PCR amplified and analyzed by direct sequencing of the products in a CLIA-approved laboratory as described previously [28] to determine other uncommon mutations that are not detected by the PIK3CA DxS kit.

### Statistical methods

#### Primary endpoint

The primary endpoint for this trial was 6-month survival, calculated as the percentage of evaluable patients alive at least 6-month postregistration. The original study design was a two-stage MinMax design with an interim analysis that was used to test whether there was sufficient evidence to determine that the 6-month survival rate was at least 35% (i.e., clinically promising) versus at most 15% (i.e., clinically inactive) [29]. This study had 91% power to detect a 6-month survival rate of 35%, with a 0.09 level of significance.

The initial trial had a planned accrual of 17 patients for the interim analysis. If at least 3 of these 17 evaluable patients lived for 6 months or more, the study would continue to a full accrual of 32 patients. Otherwise, the study would be closed early due to a lack of sufficient activity. If the study continued to full accrual, 8 or more of the 32 evaluable patients would need to live at least 6 months for the treatment to be considered promising for further study. A confidence interval for the 6-month survival rate was calculated using the exact binomial method.

When the study was negative in an unselected patient population, it was amended to enroll patients who were biomarker positive only and the plan was to enroll 7 for the stage 1 analysis and 18 patients' total (if the trial passed the stage 1 analysis). A two-stage Simon optimal design was used for this new cohort. This design had 80% power if the true 6-month survival rate was 40%, with a 9% level of significance when the true 6-month survival rate was 15%. Two- or more 6-month survivors were needed to pass the stage 1 analysis, and 5- or more 6-month survivors were needed in all 18 evaluable patients to declare saracatinib promising in the biomarker-positive patients.

#### Secondary endpoints

Secondary endpoints included adverse events, the confirmed response rate, progression-free survival, and overall survival. Adverse events were summarized in a tabular manner as the maximum grade for a given type of event for each patient. All grade 3+ adverse events are reported. Kaplan–Meier methodology was used to describe the distributions of progression-free survival and overall survival.

## Results

### Patient characteristics

Nineteen patients with gemcitabine-resistant metastatic pancreatic cancer were enrolled from four locations within the P2C network from October 2008 to January 2011 (Table 1). Of these 19 patients, 18 were enrolled to the original study and 1 biomarker-positive patient was enrolled to the new trial design. As there was only one patient enrolled to the amended trial, the data were pooled for all analyses, with a short summary of the one patient that was enrolled to the biomarker-positive portion of the study. At study registration, 8 (42%) of the patients were men, 13 (68%) had an ECOG PS of 1, and the median age was 63 years (range: 34–78). In terms of prior treatment, four (21%) received first-line gemcitabine alone, 15 (79%) received first- line gemcitabine in combination with another agent, and 7 (37%) received prior surgery related to the tumor.

**Table 1:** Baseline characteristics (*N* = 19)

Characteristic	Frequency (%)*
Age (in years), median (range)	63.0 (34–78)
Gender	
F	11 (58)
M	8 (42)
Performance status	
0	6 (32)
1	13 (68)
Prior chemotherapy	
Gemzar alone	4 (21)
Gemzar in combination with other agent	15 (79)
Prior surgery related to tumor	
Yes	7 (37)
No	12 (63)
Time to recurrence following surgery (for patients with prior surgery related to tumor)	
≤6 months	1 (14)
>6 months	6 (86)
Location of metastatic disease	
Liver only	2 (11)
Widespread	17 (89)

* Unless otherwise noted.

### Efficacy

**Figure 1:**
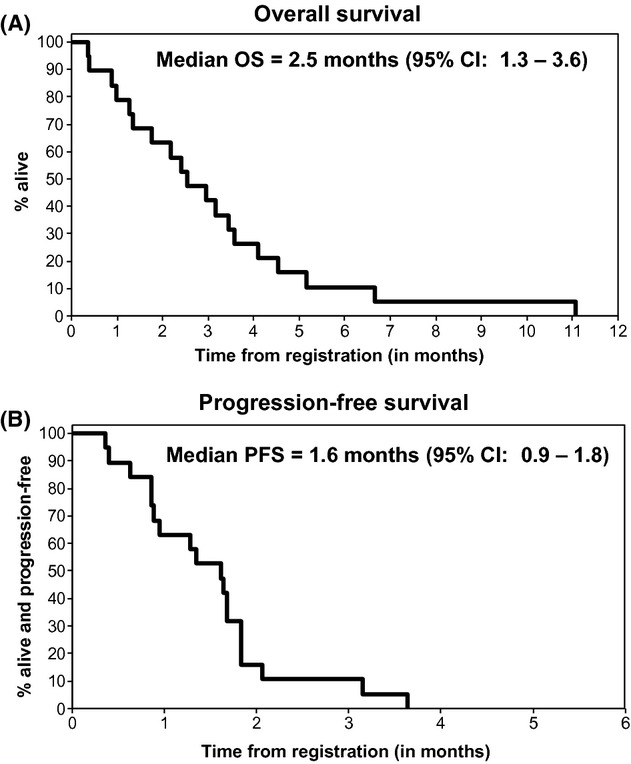
Kaplan–Meier survival curve of (A) overall survival and (B) progression-free survival.

Nineteen patients were evaluable for the outcome measures of survival, progression-free survival, and response (see Table 2). Of these 19 patients, 18 (95%) progressed and all have died. One patient died of sepsis-induced acute respiratory distress syndrome. Two (11%) of the patients survived at least 6 months (95% confidence interval [CI]: 1–33), which did not meet the criteria (three patients) for success needed to continue the trial to full accrual. The median survival (Table 2; Fig. 1A) was 2.5 months (95% CI: 1.3–3.6 months), and the median progression-free survival (Table 2; Fig. 1B) was 1.6 months (95% CI: 0.9–1.8 months). No patients had a partial or complete response to therapy, and only two patients had a best response of stable disease.

**Table 2:** Patient outcome measures (19 evaluable patients)

Clinical outcome	Statistics (95% CI*)
Survival (OS)	
6-month estimate	11 (1–33)
Median (months)	2.5 (1.3–3.6)
#alive (%)	0 (0)
Progression-free survival (PFS)	
3-month Kaplan–Meier estimate	11 (3–39)
Median (months)	1.6 (0.9–1.8)
#progression-free and alive (%)	0 (0)

* Confidence interval to the bottom of table 2.

### Dose intensity

A median of one cycle of therapy was given (range: 1–4). All patients have ended treatment. The most common reasons for ending treatment early consisted of disease progression: 12 (63%); adverse events: 3 (16%); and other reasons: 4 (21%).

### Adverse events

All 19 patients were evaluable for adverse events (Table 3 for adverse events regardless of attribution; Table 4 for events considered at least possibly related). Five (26%) of the 19 patients had a grade 3/4 toxicity, all of which were nonhematologic. Three patients had a grade 4 toxicity. One patient suffered a grade 4 aspiration (possibly related), another patient had a grade 4 gallbladder perforation (possibly related), and another patient suffered three grade 4 events (respiratory failure, hypoxia, dyspnea), all possibly related to saracatinib. No patients had a grade 5 event related to study agent.

**Table 3:** All maximum severity (grade 3/4) adverse events across all cycles of treatment (regardless of attribution)

	Incidence (%) (*N* = 19)	
	
NCI CTC category*	Grade 3	Grade 4
Hematology		
Anemia	3 (16)	0 (0)
Lymphopenia	1 (5)	0 (0)
Hepatic		
ALT	2 (11)	0 (0)
Alkaline phosphatase	2 (11)	0 (0)
AST	1 (5)	1 (5)
Bilirubin	4 (21)	1 (5)
GGT	1 (5)	0 (0)
Pulmonary		
Aspiration	0 (0)	1 (5)
Dyspnea	1 (5)	1 (5)
Hypoxia	0 (0)	1 (5)
Pneumonitis	1 (5)	0 (0)
Respiratory failure	0 (0)	1 (5)
Gastrointestinal		
Anorexia	1 (5)	0 (0)
Dehydration	1 (5)	0 (0)
Gallbladder perforation	0 (0)	1 (5)
Nausea	3 (16)	0 (0)
Vomiting	3 (16)	0 (0)
Miscellaneous		
Upper GI hemorrhage	1 (5)	0 (0)
Infection	1 (5)	0 (0)
Biliary tree infection	0 (0)	1 (5)
Blood infection	1 (5)	0 (0)
Hyponatremia	2 (11)	0 (0)
Abdominal pain	2 (11)	0 (0)
Creatinine	1 (5)	1 (5)
Thrombotic microangiopathy	0 (0)	1 (5)
Fatigue	2 (11)	0 (0)

* NCI CTC Version 3.0.

ALT, alanine transaminase; AST, aspartate aminotransferase; GGT, γ-glutamyltransferase; GI, gastrointestinal.

**Table 4:** All maximum severity (grade 3/4) adverse events across all cycles of treatment (at least possibly related to the study treatment)

	Incidence (%) (*N* = 19)	
	
NCI CTC category*	Grade 3	Grade 4
Fatigue	2 (11)	0 (0)
Bilirubin	2 (11)	0 (0)
Upper GI hemorrhage	1 (5)	0 (0)
Hyponatremia	1 (5)	0 (0)
Aspiration	0 (0)	1 (5)
Dyspnea	0 (0)	1 (5)
Hypoxia	0 (0)	1 (5)
Pneumonitis	1 (5)	0 (0)
Respiratory failure	0 (0)	1 (5)
Gallbladder perforation	0 (0)	1 (5)

* NCI CTC Version 3.0.

GI, gastrointestinal.

### Biomarker study

**Figure 2:**
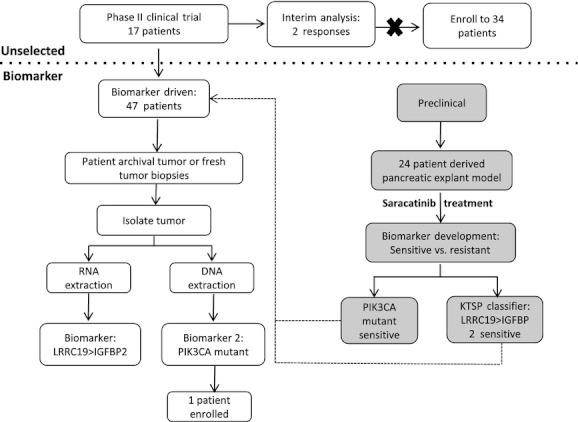
Study design: in the unselected portion, 17 patients were enrolled in the study. To move on to enroll 34 patients at least three responses were required. Only two patients made it to the 6-month endpoint. Study was amended for a biomarker-driven study. Results from our preclinical study on 24 patient-derived pancreatic explants identified the KTSP classifier LRRC19 > IGFBP2 and PIK3CA mutant as markers of sensitivity. These markers were used to screen patients for the biomarker-driven study. One patient with a PIK3CA mutation was enrolled in the study.

As the minimum number patients were not reached in the unselected portion of the study, a biomarker-driven study in selected patients was conducted to determine whether biomarkers identified from our patient-derived pancreatic adenocarcinoma explant model would be predictive of response to saracatinib (Fig. 2). Previously, we have shown that the KTSP classifier LRRC19 gene expression >IGFBP2 gene expression predicts sensitivity to saracatinib. In addition, we recently identified a second biomarker, PIK3CA mutant, to be associated (P < 0.0362) with an increased sensitivity to saracatinib (Table 5). Panc 420 had the common 542 mutation, whereas panc 410 had a novel mutation at 539. Therefore, prior to treatment, archival tumor tissue or fresh tumor biopsies were tested for the biomarker 1 (LRRC19 > IGFBP2) and/or biomarker 2 (PIK3CA mutant) to determine eligibility for the study in CLIA-approved space.

**Table 5:** Association between PIK3CA mutation and sensitivity to saracatinib

Patient ID	Saracatinib effects (TGI)	PIK3CA
P291	32	Wild type
P194	42	Wild type
JH131	44	Wild type
P410	46	Mutant
P420	48	Mutant
P185	53	Wild type
P163	54	Wild type
P281	55	Wild type
P294	61	Wild type
P140	64	Wild type
P159	71	Wild type
P198	78	Wild type
P421	85	Wild type
P287	86	Wild type
JH069	90	Wild type
P286	91	Wild type
A6L	91	Wild type
P247	93	Wild type
P215	103	Wild type
JH033	105	Wild type
P253	106	Wild type
P265	111	Wild type
JH010	137	Wild type
JH024	143	Wild type

	Saracatinib	
	
Pancreas explants	Resistant	Sensitive
PIK3CA wild type	19	3
PIK3CA mutant	0	2

*P* = 0.0362.

**Figure 3:**
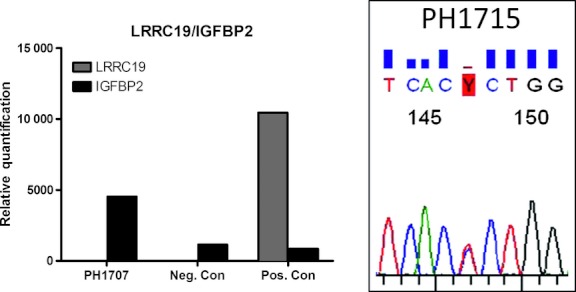
(A) Representative figure of patients archival tumor or fresh liver biopsies analyzed for LRRC19 > IGFBP2 and (B) PIK3CA mutation (3′ UTR) of PH1715 (patient enrolled in the biomarker portion of the study).

For the biomarker study, we analyzed a total of 47 patient tumor tissues from 10 different sites. As shown in Table 6, 83% consisted of archival tissue (22 blocks, 15 cut slides, 2 fine needle aspiration [FNA] slides), 15% fresh liver biopsies (RNA later and FFPE), and 2% ascites. An hematoxylin and eosin-stained slide was prepared for each of the different tissues (block, slides, or fresh core biopsy) received. The hematoxylin and eosin slide was analyzed by a board certified pathologist specializing in gastrointestinal tumors to distinguish normal cells from tumor cells and to ensure that we were mainly analyzing the biomarkers in tumor tissue. The percentage of tumor cells ranged from 0% to 100% with the majority of samples consisting of <35% tumor. Ten (21%) patients either had insufficient amount of tissue for analysis or no tumor cells present in the sample. Of the remaining 37 patients, both biomarkers were evaluated in 8 patients, the LRRC19 > IGFBP2 biomarker was assessed in 8 patients, and 21 patients were tested for PIK3CA mutations. Of note, when enough tissue was available, both biomarkers were used to determine eligibility, with LRRC19 > IGFBP2 being the priority assay due to the fact that the preclinical work establishing the assay was published. However, in cases where a limited amount of tumor tissue was available, the majority of time we examined the PIK3CA gene with the DsX kit or direct sequencing, due to concern regarding mRNA quality in archival samples. The average turnaround time from receiving the tissue to site notification of biomarker positive or negative was 4.5 business days and 6 total days. All patients tested for the gene pair were biomarker negative (LRR19 < IGFBP2) and therefore not eligible for the study (Fig. 3A). Of the 21 patients evaluated for a PIK3CA mutation, one patient was biomarker positive (PIK3CA mutant). The mutation was located in the 3′ untranslated region (UTR) of PIK3CA gene (Fig. 3B). This patient was a 64-year-old female who received gemcitabine for first-line therapy, no prior surgery, ECOG PS of 0, and had widespread metastases at baseline. Unfortunately, this patient progressed at 1.8 months and died due to this cancer after 3 months.

**Table 6:** List of tissue received for each patient, RNA/DNA concentration and biomarker assay (RT-PCR [LRRC19/IGFBP2], DsX PIK3CA kit or direct sequencing of the PIK3CA gene

				Biomarker assay		
Patient ID	Tissue	RNA concentration (ng/*µ*L)	DNA concentration (ng/*µ*L)	TSP	PIK3CA DsX kit	Direct sequencing
1	ATT	119.97	78.97	Neg	Neg	Neg
2	ATT	284.56	383.69	Neg	Neg	Neg
3	ATT	321.87	328.48	Neg	Neg	Neg
4	ATT	NP	12.66	ND	Neg	Neg
5	ATT	NP	152.13	ND	Neg	Neg
6	ATT	NP	178.3	ND	Neg	Neg
7	ATT	NP	306.15	ND	Neg	Pos
8	ATT	NP	99.29	ND	Neg	Neg
9	ATT	NP	811.99	ND	Neg	Neg
10	ATT	NP	252.98	ND	Neg	Neg
11	ATT	600.77	NP	Neg	ND	ND
12	ATT	6.05	NP	Neg	ND	ND
13	ATT	51.94	NP	Neg	ND	ND
14	ATT	398.18	256.40	Neg	ND	Neg
15	ATT	9.04	NP	Neg	ND	Neg
16	ATT	20.2	NP	Neg	ND	Neg
17	ATT	182.21	181.87	Neg	ND	Neg
18	ATT	NP	NP	N/A*	N/A*	N/A*
19	ATT	NP	NP	N/A*	N/A*	N/A*
20	ATT	NP	NP	N/A*	N/A*	N/A*
21	ATT	NP	NP	N/A*	N/A*	N/A*
22	ATT	696.5	177.29	Neg	ND	Neg
23	ATT	NP	14.6	ND	ND	Neg
24	ATT	NP	NP	N/A*	N/A*	N/A*
25	ATT	NP	9.44	ND	ND	Neg
26	ATT	NP	6.46	ND	ND	Neg
27	ATT	NP	1.39	ND	ND	Neg
28	ATT	NP	266.31	ND	ND	Neg
29	ATT	NP	4.67	ND	ND	Neg
30	ATT	NP	1	ND	Neg	ND
31	ATT	NP	1.4	ND	Neg	ND
32	ATT	NP	24	ND	Neg	ND
33	ATT	NP	109	ND	Neg	Neg
34	ATT	NP	400.6	ND	Neg	Neg
35	ATT	NP	8.67	ND	Neg	Neg
36	ATT	152.13	NP	Neg	ND	ND
37	ATT	NP	NP	N/A*	N/A*	N/A*
38	ATT	NP	NP	N/A*	N/A*	N/A*
39	ATT	NP	0	N/A*	N/A*	N/A*
40	FCLB	19.24	38.01	Neg	Neg	Neg
41	FCLB	NP	18.32	ND	Neg	Neg
42	FCLB	NP	24.88	ND	Neg	Neg
43	FCLB	10.02	NP	Neg	ND	ND
44	FCLB	14.43	NP	Neg	ND	ND
45	FCLB	7	0.68	Neg	Neg	ND
46	FCLB	NP	NP	N/A*	N/A*	N/A*
47	Ascites	NP	24.01	N/A*	N/A*	N/A*

ATT, archival tumor tissue; FCLB, fresh core liver biopsy; NP, not processed; ND, not done; N/A, not applicable; Neg, negative; Pos, positive.

* N/A, not applicable.

Due to the low frequency of biomarker-positive patients, the study was closed.

## Discussion

Saracatinib is an orally available Src/Abl inhibitor that has potent antiproliferative and antimetastatic properties in a wide range of preclinical models of solid tumors including pancreatic cancer [12]. Results from our patient-derived pancreatic adenocarcinoma explant model demonstrate that only a subset (24%) of pancreas human tumor explants have sensitivity to saracatinib and biomarkers may predict response [17]. In a phase I clinical trial of saracatinib, the maximum tolerated dose was determined to be 175 mg daily with dose-limiting toxicities including cytopenias, respiratory failure, and asthenia [30]. In addition, Src activity was shown to be inhibited in tumors and antitumor activity (confirmed stable disease) was observed in ∼14% of patients. This study, sponsored by the U.S. National Cancer Institute/Cancer Therapy Evaluation Program, was conducted by the P2C to determine the efficacy of saracatinib in second-/third-line metastatic pancreatic cancer. After failing the first-stage cut-off, it was amended to determine whether a biomarker-driven population based on our preclinical study of saracatinib was feasible and would identify patients that would derive benefit from treatment with saracatinib.

Our study showed that treatment with saracatinib was ineffective at improving the 6-month survival in an unselected patient population with metastatic pancreas cancer. Only two patients of the 18 survived 6 months and the median overall and progression-free survival was 2.5 months and 1.6 months, respectively. A recently conducted phase I/II trial of saracatinib in combination with gemcitabine in patients with advanced pancreatic adenocarcinoma showed a response rate of 26% (five stable disease with a median duration of 7.4 months and two patients had a partial response) [31]. However, this study failed to improve overall survival (median 6.2 months) when compared with gemcitabine alone. Several other phase II clinical trials with saracatinib have also shown very limited efficacy in many other solid tumors in unselected patient populations [32–36]. In particular, a phase II trial in ovarian cancer was negative when saracatinib was combined with paclitaxel, which halted further clinical development of the compound by the sponsor.

In addition to the KTSP classifier LRRC19 > IGFBP2 as a predictive biomarker of sensitivity to saracatinib in our pancreas explant preclinical model, we recently identified that a mutation in the PIK3CA gene to be associated with sensitivity to saracatinib. While the human pancreas tumor explant Panc 420 had the common mutation in exon 9 at 542, Panc 410 had a mutation at amino acid position 539. This novel mutation (C → G transition) results in a proline to arginine substitution. Both of these mutations occur in the helical domain of the p110-α subunit of PI3K and the 542 mutation has been shown to alter p85 inhibitory effects leading to an increase activation of the PI3K/AKT pathway [37]. As previously demonstrated, treatment with saracatinib in the panc410 showed a decrease in the activation of the Src pathway (p-Src, p-Fak, and p-Stat-3) as well as p-Akt [17]. In our colorectal cancer (CRC) explant model, we also found that a PIK3CA mutation was associated with increased sensitivity to saracatinib and that Src inhibition resulted in a decrease in the activation of the Src and Akt pathways [38]. Together these results indicate that Src interacts with the PI3K pathway to promote tumor growth in PIK3CA mutant tumors and that a subset of patients with this genetic alteration may show benefit saracatinib monotherapy. Further mechanistic studies are ongoing.

In the biomarker portion of the study, we enrolled one biomarker-positive patient that had a PIK3CA mutation. This mutation was located in the 3′ UTR region of the PIK3CA gene. Interestingly, in our preclinical human CRC explant model, we identified this mutation in two explants [38]. One explant exhibited the greatest sensitivity to saracatinib among 17 treated xenografts. In addition, further analysis of the functional relevance revealed that this mutation in the 3′ UTR alters the affinity for microRNA 520a and 525a, resulting in increased protein levels of the p110-α subunit of PI3K. Whether this translates to enhanced activation of the PI3K pathway remains to be determined. As a result of these findings, this patient was enrolled in the study but unfortunately progressed at 1.8 months and died at 3 months. As we also identified in CRC explants that some tumors with a PIK3CA mutation appear to be more resistant to saracatinib, it is likely that other genetic aberrations are more important at driving tumor growth in this patient's tumor. Of course, it may simply be that human tumor explant models are poorly predictive of real-life clinical cancer biology. Perhaps, examining this compound in additional models such as a patient-derived pancreas orthotopic model or a PIK3CA genetically engineered mouse model may have provided additional value as the microenvironment, especially in pancreas cancer, plays an important role in influencing treatment responses. Finally, further understanding of the genetic differences between PIK3CA mutant sensitive and mutant resistant tumors may ultimately provide a more robust biomarker, and these studies are currently underway.

Despite the negative clinical findings in this study, we have demonstrated that a multicenter biomarker testing study in advanced pancreatic cancer is feasible, especially for a DNA-based assay. There has been considerable doubt in the field regarding feasibility in biomarker-driven designs in pancreas cancer, in part, because the diagnosis is often made off of a FNA (rather than a core biopsy as in many other tumors), and the proportion of tumor cells compared with stromal elements in pancreas cancer is comparatively low. In the majority of cases, we were able to provide results with 1 week of receiving a tumor sample, many of which were submitted by community sites participating in the P2C. Although working with archival formalin-fixed samples offers some challenges mainly for RNA assays (RNA transcripts are more susceptible to degradation), using biomarkers that involve DNA analysis appears achievable.

It remains unclear why the prevalence of “positive” biomarker patients was lower than expected. On the basis of our preclinical model, we expected ∼25% of the samples we received to be positive for the LRRC19/IGFBP2 biomarker; however, of the 16 samples analyzed for this biomarker, none tested positive. Although we were meticulous in either scraping or punching tumor, it is possible that a significant amount of normal tissue may have been present when extracting RNA, and therefore, a positive signal in tumor cells may have been difficult to detect. Other factors such as the amount of time from surgical removal to tissue being placed in formalin, size of tissue placed in formalin, the time in formalin, the time before RNA isolation, and length of storage and temperature conditions all have been shown to greatly impact RNA integrity [39]. As archival tumor tissue from patients was not carefully annotated for these factors, it was difficult to assess whether these variables significantly impacted the results of this biomarker assay. Unfortunately, for these reasons, conclusions cannot be made about the LRRC19 > IGFBP2 classifier as a predictor for Src sensitivity in patients with advanced pancreas cancer in this trial. Ideally, in addition to proper FPPE procedures, future tissue acquisition will be preserved in RNA *later* or flash frozen so that biomarkers involving RNA may produce more reliable results.

With respect to the PIK3CA mutational status, we identified one patient with a PIK3CA mutation (2%) in the samples tested, which is the frequency (3%) seen in pancreas cancer [24].

In conclusion, treatment with saracatinib in an unselected population of second-/third-line pancreas cancer patients failed to improve 6-month survival compared with previous studies. In the biomarker-driven portion of the study, of the 47 patients screened only one patient was biomarker positive. This patient also failed to meet the 6-month endpoint. As the frequency of biomarker-positive (LRRC19 > IGFBP2 and PIK3CA mutation) patients was very low (<3%), the study was closed. As a result, we were unable to conclude whether enriching for a subset of patients based on a biomarker would improve the 6-month survival in this setting for selected patients. However, we demonstrated that testing samples for a biomarker-driven, multicenter study in metastatic pancreas cancer is feasible, which has important implications for drug development in pancreatic cancer. Given this negative trial in pancreas cancer as well as negative trials involving saracatinib in other malignancies, we do not believe that saracatinib monotherapy for pancreas cancer should be pursued; perhaps, combinational therapies involving a Src inhibitor may prove more beneficial in this patient population. Two pancreas cancer combinational Src inhibitor studies, gemcitabine with or without dasatinib in the adjuvant setting (NCT01234935), and gemcitabine/dasatinib in the locally advanced setting (NCT01395017), are ongoing.
